# Opportunities for Interactive Communication in Mechanically Ventilated Critically Ill Patients: A Video-Based Observational Study

**DOI:** 10.1155/2022/1885938

**Published:** 2022-07-14

**Authors:** Akiko Yamaguchi, Atsue Ishii, Haruna Fukushige, Yoshiaki Inoue, Izumi Akada, Rie Mitani, Akiko Ito, Mio Hosona, Sayaka Suga, Akiko Yamada, Yoko Arima

**Affiliations:** ^1^Department of Nursing, Graduate School of Health Sciences, Kobe University, 7-10-2 Tomogaoka, Suma-ku, Kobe, Hyogo 654–0142, Japan; ^2^Department of Information and Communications Technology, Graduate School of Engineering, Osaka University, 2-1 Yamadaoka, Suita, Osaka 565–0871, Japan; ^3^Department of Human Care Science, Graduate School of Nursing, Osaka Metropolitan University, 1-5-17 Asahimachi, Abeno-ku, Osaka, Osaka 545–0051, Japan; ^4^Department of Nursing, Higashi Takarazuka Satoh Hospital, 2-1 Nagao-cho, Takarazuka, Hyogo 665–0873, Japan; ^5^Department of Nursing, Kawasaki Hospital, 3-3-1 Higasiyama-cho, Hyogo-ku, Kobe, Hyogo 652–0042, Japan

## Abstract

**Background:**

Mechanically ventilated critically ill patients need the opportunity to communicate their physical and psychosocial concerns to nurses. However, these patients face the unique problem of lacking even the opportunity to communicate.

**Aims:**

The study aimed to describe the characteristics of communication opportunities for critically ill mechanically ventilated patients.

**Methods:**

The study was designed as a video-based descriptive observational study. Participants included seven mechanically ventilated critically ill patients at the intensive care unit, coronary care unit, or high care unit who were conscious and seven registered nurses (seven pairs). Videos were recorded continuously from 8 am to 4 pm, and the footage was then descriptively analyzed. Data collection took place between July 2019 and June 2020.

**Results:**

The total recording time was 668.0 minutes. Of these 668.0 minutes, nurses stayed in the Conversation Area of the Patient for 279.6 minutes, and of these 279.6 minutes, two-way face-to-face communication between nurse and patient occurred for 78.0 minutes. Of these 78.0 minutes, communications were started by nurses for 47.2 minutes (174 scenes) and by patients for 24.2 minutes (36 scenes). The patient-started two-way communication scenes included 37 instances of Patient-Intentional-Action that triggered the start of communication. Actions using the upper limbs were observed in 20 instances and represented the most frequently used body part. The head/face, lower limbs, or trunk were also used in some of the actions. Gestures were the most commonly used action type (14 instances). Other types included lip movement, grimace, leg flex/extension, and cough.

**Conclusions:**

We found that nurses tended to start communication more frequently than patients did and that patients demonstrated Patient-Intentional-Action with a variety of actions using various body parts. Communication opportunities for patients were created when nurses took the initiative to start communication or when they noticed and responded to the Patient-Intentional-Action. Our findings demonstrate that nurses need to recognize and always respond to Patient-Intentional-Action and to take the initiative in communicating rather than waiting for the patient to do so.

## 1. Introduction

Communication is one of the most pressing challenges when it comes to mechanically ventilated critically ill patients. Communication in clinical settings is essential [1] and improves patient outcomes [2], but patients with mechanical ventilation face unique problems with communication, such as not being understood by the nurse [3–5] or lacking even the opportunity to communicate.

Our focus in this study is on the lack of communication opportunities. Critically ill patients with mechanical ventilation typically have physical problems such as pain, dyspnea, thirst, or sleeplessness that need to be communicated [6–8], and they also experience psychosocial problems such as anxiety or fear triggered by receiving invasive treatment in critical care settings [8, 9]. It is essential that patients have the chance to communicate these physical and psychosocial concerns to nurses, but they may not have access to such opportunities. The lack of opportunities to communicate is therefore a serious issue that needs to be resolved.

Prior research has indicated that patients experience a lack of opportunities to communicate with nurses. Noguchi and Inoue [10] showed that patients had no chance to communicate because nurses were not aware that they wanted to, even though the patients were signaling. Yamaguchi et al. [11] reported that patients experienced being left alone without nurses being aware of their communication cues. Karlsson et al. [12] found that patients experienced feelings of neglect and of the nurses being absent, as nurses did not speak to them or stay close enough to notice their signals. Wallander Karlsen et al. [13] identified attention-seeking actions performed by patients and noted that while nurses responded immediately to such cues, the responses were sometimes too late. These studies clearly indicate that patients want to communicate with nurses but lack the opportunities to do so.

Previous studies [10–13] have also identified the lack of communication opportunities as a typical experience when wearing a mechanical ventilator. However, there have been very few studies [10–13] that focus on communication opportunities themselves for patients and describe them in detail. As such, it remains unclear whether the patients have communication opportunities, what kind of opportunities these may be, and how long these opportunities may last. It is also not clear what actions patients use as cues to indicate the desire for communication with nurses and how often these actions are performed. To answer these questions accurately, it is necessary to obtain continuous observational data over a long period of time, rather than partial observational data for just a few hours while the patient is on the ventilator, and to analyze this data and describe it in detail. Such effort would enable us to examine nursing practices more thoroughly to ensure that patients have the opportunity to communicate.

In this study, we aimed to describe the characteristics of communication opportunities for critically ill mechanically ventilated patients with respect to the following research questions: (1) What is the frequency and duration of communication between patient and nurse? (2) What actions do patients take to signal a desire to start communication? Communication opportunity is defined here as a two-way face-to-face interaction in which a person verbally or nonverbally conveys their thoughts and feelings to another.

## 2. Methods

### 2.1. Study Design

The study was designed as a video-based descriptive observational study.

### 2.2. Setting

We collected the data from three units of two hospitals in cities located in western Japan. One hospital was a 240-bed general hospital, where four of the beds were located in the high care unit (HCU). The other hospital was a 150-bed cardiovascular hospital, where eight of the beds were in the intensive care unit (ICU), and four were in the coronary care unit (CCU).

Patient beds were located on an open floor, on a semiopen floor, or in a single-occupancy room. On the open and semiopen floors, the beds were separated by curtains or walls, respectively. The single-occupancy room was a separate private room. The HCU had an open floor and one single-occupancy room, the ICU had an open floor and a semiopen floor, and the CCU had an open floor.

The patient-to-nurse staffing ratios per 24-hour day were 2 : 1 in the ICU and CCU and 4 : 1 in the HCU. The number of patients assigned to each nurse was typically small during the day shift and larger during the evening and night shifts. Nurses were responsible for handling various aspects of the patients' care, such as performing physical examinations, administering medications, assisting in medical care, collecting blood for blood tests, giving sponge baths, performing oral care, helping with family care, transporting the patient, and keeping records.

### 2.3. Participants

Mechanically ventilated critically ill patients and the registered nurses assigned to them participated in the study. Patients were considered eligible if they were mechanically ventilated with intubation or tracheostomy, were being treated at the ICU, CCU, or HCU, had a Glasgow Coma Scale (GCS) score of E3VTM6 or higher, and (in the case of sedation) had a Richmond Agitation Sedation Scale (RASS) score within the range of −1 to 1. Eligibility for the nurses was restricted to registered nurses on the day shift assigned to the patients who consented to participate in this study.

### 2.4. Data Collection

Data collection took place between July 2019 and June 2020. We utilized video recording for the data collection because it enabled us to observe all the actions taken by patients and nurses and to access the data repeatedly. Observing using video recording is known to be a useful and powerful data acquisition tool [14, 15]. Video recordings can accurately record the complex nature of nursing phenomena [14] and allow multiple researchers to scrutinize the data during the process of analysis [15].

Fixed-point video cameras were placed at the head and foot of each patient's bed. The video cameras were GoPro HERO6 Black edition (GoPro, Inc.), and the records stored 2.4 K and 30 frames per second. We recorded videos from 8 am to 4 pm for only one day during the mechanical ventilation period. We planned the video recording during the day shift because we wanted to capture as many communication opportunities as possible. Shift schedules at the hospitals were organized as two-shift or three-shift rotations. The day shift began at around 8 am in both rotations. Night shifts in the two-shift rotation and evening shifts in the three-shift rotation began at around 4 pm. In consideration of the shift schedules, we decided on the recording time of 8 am to 4 pm and kept recording continuously over this span except for physical examinations of the chest or abdomen, excretion care, sponge baths, family visits, and nurse break times. If the endotracheal tube was extubated before 4 pm, we ended recording at the time of extubation. The researcher was always at the units where the data were collected so that the recording could be stopped at any time if requested. However, the researcher stayed at a distance from the participants and did not speak to them at any time during the recording.

Prior to starting, we collected information about the patients and nurses, as shown in Tables 1 and 2. The patient information was gleaned from electronic medical records and included age, diagnosis, treatment, days on mechanical ventilation, airway, and any sedatives. We evaluated the GCS status and RASS status by observing patient-nurse interactions at the beginning of data collection. Information on the nurses included age and years of experience as a nurse and as a critical care nurse obtained through interviews.

**Figure 1:**
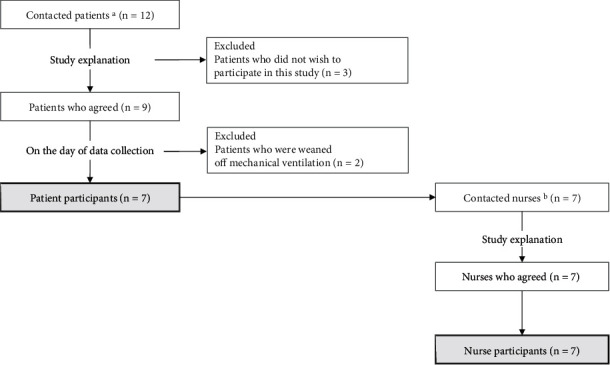
Flowchart of participant recruitment. (a) Contacted patients: patients were already equipped with mechanical ventilation or planned to use mechanical ventilation postoperatively. (b) Contacted nurses: day shift nurses assigned to patients who participated in the study.

Seven mechanically ventilated critically ill patients in the ICU, CCU, or HCU who were conscious and seven registered nurses on the day shift assigned to the patients participated in the study (seven pairs). Each patient and nurse participated in the study for only one day.  Figure 1 shows the flow of recruiting participants. Patients who were already equipped with mechanical ventilation or planned to use mechanical ventilation postoperatively were asked in advance to participate in the study, and consent was obtained. However, if the patients had already been weaned off the ventilator on the date of data collection, they were excluded from the study even if consent had been obtained. The Verbal Response, one element of GCS, was not testable in all patients because of intubation or tracheostomy (Table 1). However, they could respond to queries such as those containing their name. Five patients were on the open floor, and two were on the semiopen floor. Of the seven nurses, six were assigned to one patient on the day of data collection, and one was assigned to two patients.

### 2.5. Ethical Considerations

The ethics committee of the Graduate School of Health Sciences, Kobe University, approved this study (approval number: 682), as did the ethics committees of Higashi Takarazuka Satoh Hospital (approval numbers: 1–6) and Kawasaki Hospital (approval number: 1-4-1).

We contacted patients who planned to use mechanical ventilation and those who were already mechanically ventilated to recruit study participants. All patients who planned to use mechanical ventilation provided written informed consent. All who were already mechanically ventilated gave oral informed consent, and their family provided written informed consent. We explained the study outline to unit nurses in advance. On the day of data collection, we contacted the eligible nurses again to explain the study and obtain written informed consent, which all of the nurses gave. Healthcare professionals included medical doctors, physiotherapists, or nurses who provided care in conjunction with the assigned nurses. They were excluded from the analysis if they did not participate in communication with patients and nurses who consented to participate in this study; hence, only oral informed consent for video recording was obtained on the day of data collection. However, in cases where healthcare professionals were participating in communication between the patients and the nurses, written informed consent was also obtained.

The recorded data were stored on external media in a password-protected file and kept in a locked locker. Only researchers involved in the analysis were allowed to access the data, and we used a dedicated research computer that was not connected to them through the Internet.

### 2.6. Data Analysis

We first classified the recorded data (see Section 2.6.1) and then analyzed it to identify the frequency and duration of communication between patients and nurses (see Section 2.6.2). We also investigated what types of actions patients took to signal a desire to start communication and how many times the patients performed those actions (see Section 2.6.3). The analysis results were reported and discussed at regular meetings attended by multiple researchers who were licensed registered nurses with clinical experience. ELAN version 5.9 was used for annotating the video.

#### 2.6.1. Classification of Recorded Data

**Figure 2:**
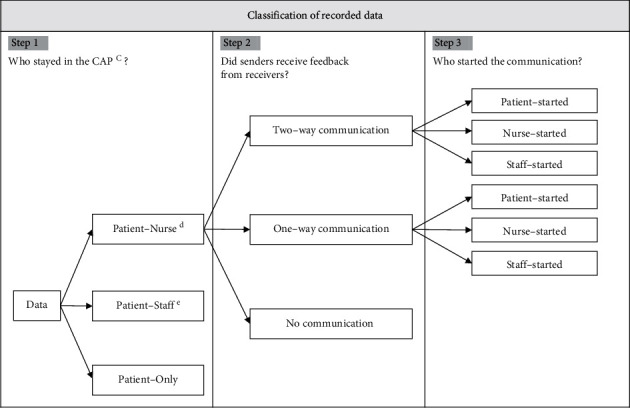
Flow diagram for classification of recorded data. (c) CAP: Conversation Area of the Patient. A space approximately 150 cm to the left and right from the center of the bed on which a patient is lying and approximately 50 cm above the headboard. (d) The nurse in Patient-Nurse refers to nurses assigned to the patients who participated in the study. Some Patient-Nurse scenes included additional healthcare professionals such as medical doctors, physiotherapists, medical engineers, or nurses who provided care in conjunction with the assigned nurses. (e) The staff in Patient-Staff refers to healthcare professionals and does not include nurses assigned to the patients who participated in the study.

As shown in Figure 2, we performed three steps (Steps 1, 2, and 3) to classify the recorded data.

**Step 1 .**

Who stayed in the Conversation Area of the Patient?

**Figure 3:**
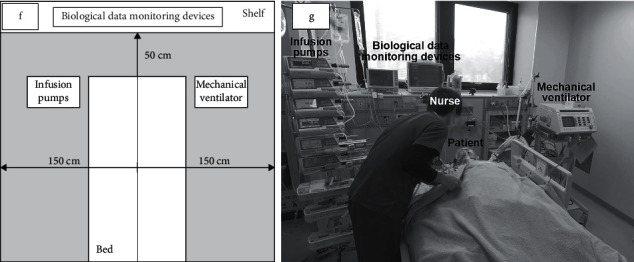
Conversation Area of the Patient (CAP). (f) Schematic of CAP. (g) Photograph of CAP.

We classified data into three types based on who was in the Conversation Area of the Patient (CAP). Hashimoto et al. [16] reported that an interpersonal distance of 150 cm or less without conversation is uncomfortable, so we defined CAP here as a space approximately 150 cm to the left and right from the center of the bed on which a patient was lying and approximately 50 cm above the headboard (Figure 3). We set the space above the headboard to 50 cm because there was a shelf at the back of the headboard, leaving only approximately 50 cm of space.

First, we extracted data in which the assigned nurses were in the CAP and classified them as Patient-Nurse scenes. Patient-Nurse scenes always included the patients and their assigned nurses. Moreover, the medical doctors, physiotherapists, medical engineers, or nurses who provided care in conjunction with the assigned nurses were included in some Patient-Nurse scenes.

Second, we extracted data in which healthcare professionals were in the CAP and classified them as Patient-Staff scenes. Patient-Staff scenes always included patients and healthcare professionals such as medical doctors or physiotherapists but did not include the assigned nurses. We classified scenes with no one in the CAP except the patient as Patient-Only scenes.

One Patient-Nurse scene was extracted as a single continuous scene from the time when a part of a nurse's body entered the CAP to when the entire nurse's body left the CAP. The Patient-Staff scenes were extracted in the same way.

**Step 2 .**

Did senders receive feedback from receivers?

We classified the Patient-Nurse scenes into three categories based on whether senders received feedback from receivers.

First, we extracted the communication-related actions of patients, nurses, and healthcare professionals. Communication-related actions in patients were all head, upper limb, lower limb, trunk, and facial movements, without considering the intention of communication. However, we excluded eye movements (opening/closing eyes and gazing). The participants in this study included postoperative patients, some of whom had eyelid edema and were unable to open their eyes, which meant that gazing could not be observed. Therefore, eye movements were excluded because it was not possible to extract eye movements under identical conditions from all participating patients. Communication-related actions in nurses and healthcare professionals were utterances to patients. In this study, we dealt only with utterances to examine the times that nurses or healthcare professionals communicated with patients using vocal language.

**Figure 4:**
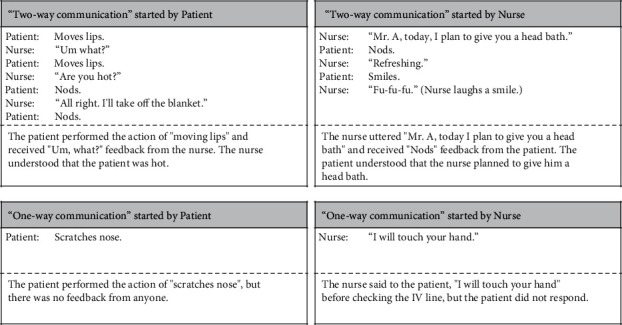
Examples of two-way and one-way communication scenes. The second row of each box shows the actual communication between the patient and the nurse. In the boxes showing one-way communication, only the actions of the patient or the nurse are displayed. The third row of each box shows the explanation of scenes.

Second, we identified two-way or one-way communication by comparing the communication-related actions of patients, nurses, and healthcare professionals. Two-way communication was a single continuous scene that started when the sender performed the first action and received feedback on it and ended when the receiver understood the sender's thought or feeling. Regarding the understanding of thoughts or feelings, we repeatedly observed communication-related actions of patients, nurses, and healthcare professionals on the video footage that demonstrated the thoughts and feelings of receivers were understood. However, there were also scenes where the receivers did not ultimately understand. For example, in one scene, a nurse eventually said to a patient, “I am sorry, but I can't understand what you're telling me.” Examples of two-way communication scenes are presented in Figure 4.

One-way communication was a single continuous scene that started when the sender began the action and ended when the action finished. There was no feedback on that action. In these scenes, we did not consider whether the actions were intentional or not. Examples of one-way communication scenes are provided in Figure 4.

We classified any scenes that did not correspond to either two-way or one-way communication scenes as “no-communication.” In no-communication scenes, patients, nurses, and healthcare professionals did not perform any communication-related actions.

**Step 3 .**

Who started communication?

We classified each two-way and one-way communication scene into one of three categories based on who started the communication: when a patient took the first action, when a nurse took the first action, and when a healthcare professional took the first action.

#### 2.6.2. Frequency and Duration of Communication between Patients and Nurses

We calculated the total duration of each of the scenes classified in Section 2.6.1, namely, Patient-Nurse scene, Patient-Staff scene, and Patient-Only scene; two-way communication scene, one-way communication scene, and no-communication scene; and patient-started, nurse-started, and staff-started two-way and one-way communication scene. We also counted the number of two-way communication scenes started by patients or nurses. Note that all scenes were calculated on the order of milliseconds, but we use minutes for the discussions in this paper.

#### 2.6.3. Types and Frequency of Actions and the Body Parts Used for Those Actions

Patient-Intentional-Action, which is an intentional action taken by the patient to signal a desire to start communication in a patient-started two-way communication scene, was identified as follows.

First, from the two-way communication scenes started by patients, we extracted the first actions performed by patients in each scene as the Patient-Intentional-Action.

Second, we classified Patient-Intentional-Action into four categories: (1) head/face, (2) upper limbs, (3) lower limbs, and (4) trunk, depending on which body part was used to perform the action. We then calculated the frequency of actions that were performed using each part of the body.

Finally, we identified Patient-Intentional-Action types inductively by comparing the actions performed using each part of the body and classifying similar actions into the same category. We repeatedly reviewed the video to classify the actions and then counted the number of action types and the number of actions for each type.

## 3. Findings

### 3.1. Frequency and Duration of Communication Opportunities between Patients and Nurses

The total recording time was 668.0 minutes (see Table 3). The longest record was 194.8 minutes, and the shortest was 38.0 minutes.

**Figure 5:**
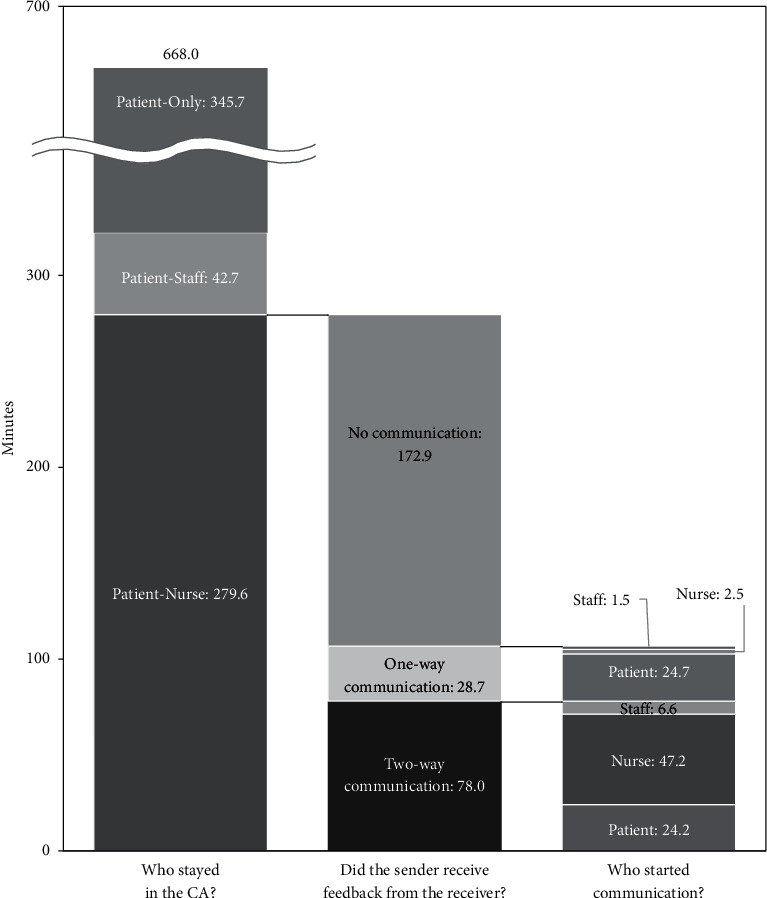
Communication opportunities between patients and nurses and their durations.

Of the 668.0 minutes of footage, Patient-Nurse scenes accounted for 279.6 minutes and Patient-Only scenes for 345.7 minutes (Figure 5).

Of the 279.6 minutes of Patient-Nurse scenes, two-way communication scenes accounted for 78.0 minutes and one-way communication scenes for 28.7 minutes (Figure 5). There was also a total of 172.9 minutes of no-communication scenes (Figure 5).

Of the 78.0 minutes of two-way communication scenes, those started by patients accounted for 24.2 minutes (36 scenes) and those by nurses for 47.2 minutes (174 scenes) (Figure 5). Of the 28.7 minutes of one-way communication scenes, those started by patients accounted for 24.7 minutes and those by nurses for 2.5 minutes (Figure 5).

### 3.2. Types and Frequency of Patient-Intentional-Actions and the Body Part Used for Those Actions

**Figure 6:**
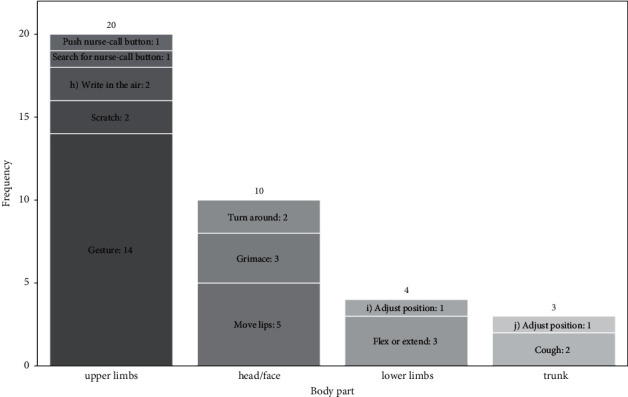
The types of actions patients take to make nurses aware of communication intent (*n* = 37). (h) Write in the air with a finger. (i) Adjust the position by abduction, adduction, lateral rotation, or medial rotation of lower limbs. (j) Adjust the position by lifting back or shoulder.

We extracted 37 Patient-Intentional-Actions from the 36 two-way communication scenes started by patients (there was one extra action because one patient performed two actions at the same time). Of the 37 actions, patients performed 20 using the upper limbs and ten using the head or face (Figure 6).

We categorized the 37 Patient-Intentional-Actions into 12 action types (lip movement, grimace, gesture, write in the air, flex or extension of lower limbs, cough, etc.). The most common action was gesture, which we observed 14 times. Examples of gestures in our study include the patient pointing to the intubation tube, the patient beckoning to a nurse, and the patient miming the action to drink water. Lip movement to imitate speech was observed five times and was only used by the tracheostomy patient. Ten types of action other than gesture and lip movement were observed one to three times each (Figure 6). Pushing the nurse-call button, searching for the nurse-call button, adjusting the position of the lower limb, and adjusting the position of the trunk were observed once each.

## 4. Discussion

Our analysis clarified the frequency and duration of communication between patients and nurses and the types and frequency of Patient-Intentional-Actions. To date, few studies have described in detail the frequency and duration of communication between patients and nurses based on data obtained from video recordings. In addition, while previous studies [17–19] have shown which communication methods patients use and how much they use them between the beginning and end of communication, none have examined Patient-Intentional-Actions as a trigger to start communication, which was the focus of our study. To our knowledge, the results of our study represent key findings that provide suggestions for nursing practices to secure better communication opportunities for patients. Our main findings are as follows.

Nurses tended to start two-way communication more frequently than patients did, which suggests that nurses take the initiative and create opportunities for communication with patients. This finding is consistent with reports that communication exchanges are most often started by nurses [17]. Patients have recently been mechanically ventilated without sedation or with light sedation, as recommended by “Clinical practice guidelines for the prevention and management of pain, agitation/sedation, delirium, immobility, and sleep disruption in adult patients in the ICU” [20], which has enabled nurses to communicate with them more interactively than ever before. Laerkner et al. [21] reported that mechanically ventilated patients can initiate, direct, and participate in communication from the first days of critical illness. On the other hand, Wallander Karlsen et al. [13] found that patients struggle to perform the actions required to initiate communication. These patients are often seriously ill and attached to many devices, which makes it difficult for them to take actions that show their intention to communicate, that is, Patient-Intentional-Action. Therefore, nurses should take the lead and initiate communication rather than waiting for Patient-Intentional-Action from patients who have difficulty performing such actions. These practices would ideally reduce the physical burden on patients and enable their communication needs to be met in advance.

However, one-way communication in which the patients did not respond to the nurses' utterances was also identified. The reasons for which patients did not respond to the nurses' utterances were unclear, but it might be that patients were not free to move their bodies due to pain, fatigue, edema, sedation, or medical equipment, they could not hear the nurses, or they were resting. Alasad and Ahmad [22] reported that nurses sometimes forget to communicate with patients when the patients are unconscious and unresponsive. However, Lawrence [23] indicated that patients listen, understand, and respond emotionally to what nurses have said, even when they appear to be unconscious. The patients may not be able to respond visibly for whatever reason but might hear and understand the nurses' words and respond in their own way. Nurses are responsible for communicating with patients whether they respond or not, so it is important for them to keep up their communication effort.

Patient-Intentional-Actions were performed the most frequently using patients' upper limbs. This suggests that even patients who are critically ill, fit with many devices, or have difficulty moving their bodies can still use their upper limbs to perform actions that indicate their intent to communicate. Therefore, nurses should consider ways of making it easier for patients to use the upper limbs as a means of communication. For example, medical devices should be placed on the nondominant hand if possible so that the dominant hand can be used freely as a means of communication. We also found that some Patient-Intentional-Actions were performed using either the head/face, lower limbs, or trunk in addition to upper limbs. These results are similar to those of Wallander Karlsen et al. [13], who reported that patients mostly used their lips, hands, or legs to initiate the first contact with nurses. Patients naturally take actions using their body parts to demonstrate their communication intent when they cannot use their voice. It is essential that nurses carefully observe the upper limbs, but they must also observe the head, face, lower limbs, or trunk so as not to miss any actions that are cues to start communication and to get the patients to the starting line of communication with nurses.

While it is important to carefully observe actions that indicate a patient's desire to communicate, this study contributed insight into why attention to the patients' actions may be lacking. Specifically, it might be because nurses are not within adequate observation distance of patients' actions. In fact, we found that “Patient-Nurse” scenes were shorter than “patient-only” scenes. This is not to say that nurses spent too little time in the CAP, as they were often performing a variety of tasks for patients (e.g., IV preparation, consulting with other healthcare professionals, and writing nursing record entries) that could not be performed within the CAP. However, paying attention to patients' actions logically requires that nurses be in a position to observe these actions. Another finding in this study is that there were many one-way communications in which the nurses did not respond to the patients' actions. This implies that nurses missed several chances to pick up on actions as communication cues even though they were in the CAP. Although there are times when the operation of infusion pumps, mechanical ventilators, or biological data monitoring devices demands careful attention to those devices, which might prevent the nurses from paying attention to the patients, the nurses should still be required to constantly observe the patient's actions when in the CAP. For example, they should stand in a place where they can see the patients' actions and must be careful not to turn their backs on patients. We believe that if nurses perform more tasks in the CAP and devise ways to observe patients' actions, it may make it easier for them to notice Patient-Intentional-Actions and thus be less likely to miss patient behavior. However, we have not yet been able to verify whether the patient's action during one-way communication actually indicates a willingness to communicate. Further verification is required to determine whether there are any specific actions that nurses should respond to. Such verification will reveal patient actions that nurses often overlook and suggest nursing practices for creating communication opportunities without missing those actions.

In this study, we focused on Patient-Intentional-Actions taken by patients to inform nurses of the intention to start communication. However, from a different perspective, these Patient-Intentional-Actions could be considered as actions in which the nurses recognized that the patients' actions were made with the intention to start communication, and it was the nurses' responses that created the opportunities to communicate. In other words, whether or not an opportunity for communication occurs depends on how the recipient of an action handles that action.

In our study, we found that nurses recognized gesture, lip movement, grimace, adjusting the position of the lower limb, or adjusting the position of the trunk as Patient-Intentional-Actions showing the intention to initiate communication. We clarified that the nurses noticed the intention to start communication in a variety of actions and created communication opportunities for patients accordingly. It has been reported that nurses must interpret the patient's facial expressions and body language to determine what action to take [12], but it is also true that nurses may perceive the patient's physical movements not as an initiative to communicate but as restlessness or agitation, both of which are common in the ICU [13]. It is thus necessary for nurses to interpret whether or not the patients' actions are signals to start communication. However, this might prove difficult when, for example, the patient simply moves their body. In such cases, it is recommended that nurses speak to the patients to confirm. If the nurses call out and the patients have no intention of initiating communication, the patients will say that everything is fine. Sometimes, the patients might tell nurses something they were planning to tell them later. In any case, it is important for nurses to always respond to Patient-Intentional-Actions to determine what they are.

Interestingly, we found that patients used the nurse-call button just once, even though this was the easiest way to signal nurses. Actions with sound are more noticeable to nurses than actions without sound, such as beckon and grimace, which suggests that actions with sounds should be utilized to make the nurses more aware of the patients' intentions and thereby create opportunities for communication. An earlier study pointed out that patients in the ICU needed to have some kind of sound-activating device nearby so that they could get the attention of a healthcare professional quickly [13]. We recommend preparing a nurse-call device that patients can always hold in the hand and press when needed, such as a palm-sized device with a call button at the end of a cord. Nurses should encourage patients to utilize these devices because doing so can create easier communication opportunities for both patients and nurses. It is also necessary to develop new equipment that can give a signal when the patients want to talk to the nurses without much effort.

Finally, in terms of future research that would address the lack of communication opportunities for critically ill patients on mechanical ventilators, intervention and experimental studies should be conducted to build evidence of nursing practice. Prior studies [10–13] have confirmed that patients experience a lack of communication opportunities and have proposed nursing practices to address the problem. The present study similarly suggests several nursing practices that can be implemented and evaluates the nursing practices indicated in these studies in clinical settings. For example, it would be helpful to conduct an intervention study to examine whether having nurses spend more time in CAP increases their awareness of patient communication cues and the time they spend communicating with them.

## 5. Strengths and Limitations

We think that video recording is one of the best techniques for observational study, as this approach makes it possible to collect data that is difficult to capture or that would otherwise be missed in the field. For example, we were able to capture even the smallest actions, such as movements of a patient's fingertips. In addition, researchers could repeatedly reexamine the same scenes during the analysis process. For example, in situations where it was difficult to determine how to classify a certain patient action, multiple researchers viewed the video footage, held discussions, and finally decided on it. Haidet et al. [24] reported that video recordings can be replayed any number of times and provide a high degree of reproducibility when measuring observations. In this way, utilizing the video recording technique enabled us to ensure reliable data collection and analysis.

One of the unique contributions of this work is our observation and classification of Patient-Intentional-Actions, which will be useful for the construction of a monitoring system that automatically detects these actions. Moreover, as these Patient-Intentional-Actions pertain to the actions that nurses sensed and responded to, they can also be utilized for a system equipped with a program that mechanically monitors Patient-Intentional-Actions and signals the nurses when those actions occur.

However, our study has some limitations.

First, we collected only seven cases at two hospitals, and more cases at multiple hospitals should be collected in the future. More cases would provide further suggestions and allow for the transfer of knowledge and methods of communication with mechanically ventilated patients to clinical and educational settings.

Second, we only considered utterances as communication-related actions of the nurses, as the time nurses communicated with patients was determined on the basis of their usage of vocal language. However, communication can include many actions other than utterances, for example, watching and touching. In this study, if watching and touching occurred without the nurses making any utterances, they were classified as no-communication scenes, but they could be considered as communication scenes depending on the focus of the study. Examples would include research on how often nurses use touch during communication or what part of the patient the nurse sees and speaks to during communication. Future work should extract and analyze such communication-related actions in accordance with the research focus. However, it is difficult to measure watching and touching by video recording alone, so data collection using devices that can trace the nurses' line of sight or sense of touch should be used in combination with video recording. In addition, the effects of seeing and touching during communication with patients should be analyzed from both quantitative and qualitative points of view.

Finally, our study was limited to a quantitative examination of patients' communication opportunities. Haidet et al. [24] have pointed out that video recording data can show what happens in real time but may lack important contextual data. Future research should utilize qualitative methods (e.g., patient interviews in combination with video recording) to analyze the interactions between patients and nurses and clarify what the opportunities would mean to patients.

## 6. Conclusion

The results of our video-based descriptive observational study of the communication opportunities for mechanically ventilated critically ill patients showed that nurses created communication opportunities more frequently than patients did, that patients performed various types of Patient-Intentional-Action using either the upper limbs, head/face, lower limbs, or trunk, and that the nurses responded to these actions. Our findings suggest that communication opportunities are created when nurses take the initiative to talk to patients or recognize and always respond to Patient-Intentional-Actions. We recommend that nurses take the initiative to talk to patients rather than waiting for them to initiate, carefully observe Patient-Intentional-Action so as not to miss it, and respond to Patient-Intentional-Action whenever it is observed.

## Figures and Tables

**Table 1:** Characteristics of patients (*n* = 7).

Characteristic	Value
Age (years)	
Mean	71.3
Range	43–88

Sex	
Male	4
Female	3

Diagnosis/treatment	
Surgical	6
Medical	1

Airway	
Intubation	6
Tracheostomy	1

Unit	
ICU	5
CCU	1
HCU	1

Days on mechanical ventilation	
Mean	5.9
Range	1–23

GCS^*∗*^	
E3VTM6	4
E4VTM6	3

Sedation	
No sedation	4
Propofol	2
Dexmedetomidine	1

RASS^*∗∗*^	
Score 0	2
Score −1	1

*Note*. ^*∗*^GCS: Verbal Response, one of the elements of the GCS, could not be tested in all patients because of intubation or tracheostomy. Therefore, Verbal Response was denoted as VT: Verbal Tube. ^*∗∗*^RASS: the RASS status was evaluated only in three sedated patients.

**Table 2:** Characteristics of nurses (*n* = 7).

Characteristic	Value
Age (years)	
Mean	31.0
Range	24–40

Sex	
Male	3
Female	4

Years of experience	
As a nurse	
Mean	5.5
Range	1.3–11.0

As a critical care nurse	
Mean	3.0
Range	0.9–6.0

**Table 3:** Characteristics of video footage (*n* = 7).

Characteristic	Value
Total	668.0
Mean	95.4
Range	38.0–194.8

*Note*. Unit: minutes.

## Data Availability

The data are not publicly available due to privacy or ethical restrictions.
